# Breaking the curse of dimensionality to identify causal variants in Breeding 4

**DOI:** 10.1007/s00122-018-3267-3

**Published:** 2018-12-13

**Authors:** Guillaume P. Ramstein, Sarah E. Jensen, Edward S. Buckler

**Affiliations:** 1 Institute for Genomic Diversity, Institute of Biotechnology, Cornell University, 175 Biotechnology Building, Ithaca, NY 14853 USA; 2 Section of Plant Breeding and Genetics, Cornell University, Ithaca, NY 14853 USA; 3 United States Department of Agriculture, Agricultural Research Service, Ithaca, NY 14853 USA

## Abstract

In the past, plant breeding has undergone three major transformations and is currently transitioning to a new technological phase, Breeding 4. This phase is characterized by the development of methods for biological design of plant varieties, including transformation and gene editing techniques directed toward causal loci. The application of such technologies will require to reliably estimate the effect of loci in plant genomes by avoiding the situation where the number of loci assayed (*p*) surpasses the number of plant genotypes (*n*). Here, we discuss approaches to avoid this curse of dimensionality (*n *≪ *p*), which will involve analyzing intermediate phenotypes such as molecular traits and component traits related to plant morphology or physiology. Because these approaches will rely on novel data types such as DNA sequences and high-throughput phenotyping images, Breeding 4 will call for analyses that are complementary to traditional quantitative genetic studies, being based on machine learning techniques which make efficient use of sequence and image data. In this article, we will present some of these techniques and their application for prioritizing causal loci and developing improved varieties in Breeding 4.

## Past and current trends in plant breeding

**Fig. 1:**
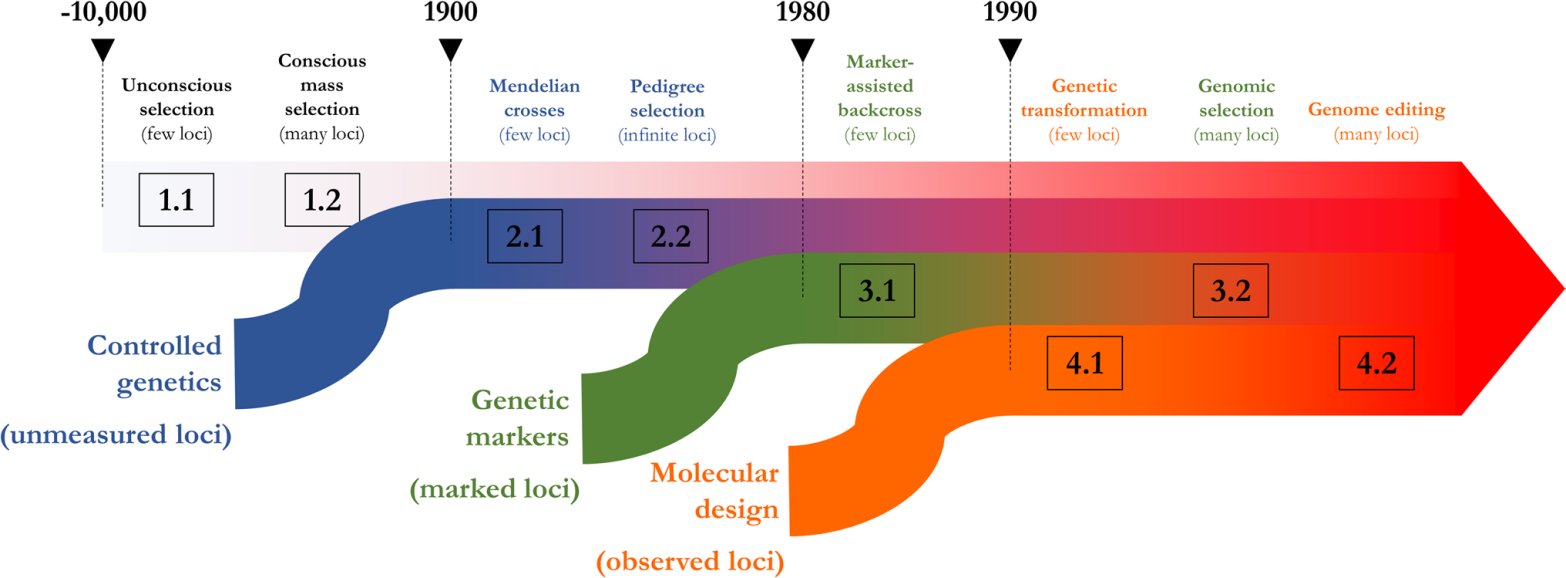
Timeline of plant breeding phases. Breeding 1, selection with unknown loci; Breeding 2, selection by controlled crosses; Breeding 3, marker-assisted selection; Breeding 4, ideotype-based selection and transformation

This perspective article considers technologies that have contributed to the evolution of plant breeding and focuses on innovations that will shape its future, amid a growing world population and changing climate. Since the Neolithic revolution, plant breeding has gone through distinct phases, separated by radical transformations. Within each of these major technological phases, we see a pattern that we would now call a shift from monogenic to polygenic focus. While it is tempting to believe this pattern is the product of greater understanding, it is most likely the product of how adaptation proceeds, as suggested by the Fisher–Orr geometric model (Fisher 1930; Orr 1998). A population evolving toward a new evolutionary optimum is more likely to substitute large-effect mutations in the first generations, while subsequent generations continue to fix smaller-effect mutations. Over time, it generally takes orders of magnitude more small effects to climb the population’s adaptive peak (Orr 2005). So, as new evolutionary optima have been set by environmental changes (e.g., domestication, management practices, biotic or abiotic pressures) or major shifts in genetic background, plant breeders have selected better-adapted genotypes while pursuing faster genetic gains through technological innovations and scientific breakthroughs. As these discoveries marked transformations in plant breeding, different phases have built upon one another, each time expanding the set of tools available for making progress (Fig. 1).

The first phase, which we refer to here as Breeding 1, accounts for the first 10,000 years of crop improvement. Early plant breeders domesticated useful species and unintentionally promoted domestication traits, such as reduced shattering in maize (Stitzer and Ross-Ibarra 2018) and grain composition in rice (Kovach et al. 2007) (Breeding 1.1). These domestication traits likely consisted of a few major genes controlling dramatic changes in crop physiology, as is suggested by genetic studies comparing maize to its ancestor teosinte (Doebley et al. 1990; Briggs et al. 2007). Early domestication phases were followed by long periods of deliberate selection for increased performance, probably involving mass selection for traits with a complex polygenic basis, such as plant environmental adaptations and yield (Breeding 1.2).

The second major phase in plant breeding, Breeding 2, began much later, in the early- and mid-twentieth century, and was characterized by the understanding and implementation of Mendelian genetics (Mendel 1866; De Vries 1900; Correns 1900) as well as the development of methods to account for environmental variability by experimental design (Fisher 1935; Cochran and Cox 1950). These advances allowed introgression of important genes for modern agriculture (Biffen 1905), including disease resistance genes under the gene-for-gene hypothesis (Flor 1971) and dwarfing genes in wheat and rice which enabled the Green Revolution (Hedden 2003) (Breeding 2.1). This phase was also marked by the development of familial crosses brought about by critical innovations in statistical science (Breeding 2.2). The genetic theory of resemblance between relatives initiated by Fisher (1919) and Wright (1921) supported impactful breeding schemes (e.g., sib mating and selfing) which resulted in higher selection gains by increased heritability at the family level (among-family selection being more accurate than mass selection) and hybrid vigor by inbred crosses (making use of genomes’ complementarity) (Bernardo 2002). Furthermore, a theoretical framework to predict genetic gains was introduced by the “breeder’s equation,” attributed to Lush (1937) (Turelli 2017). Finally, linear mixed models developed by Henderson et al. (1959) provided a valuable tool to account for familial relationships in the prediction of genetic merit, initially in animal breeding and, much later, in plant breeding (Panter and Allen 1995).

The third phase in crop improvement, Breeding 3, added genomics to the breeder’s toolbox. Marker-assisted breeding made it possible to consider both phenotype data on agronomic traits and genotype data from DNA information. Effects of polymorphisms could finally be estimated, and causal loci previously unmeasured in Breeding 2 became exposed, though indirectly, by association between phenotypes and genetic markers physically linked to causal loci. The first breeding applications of genomics consisted in mapping quantitative trait loci (QTL) in experimental populations, using genetic markers such as restriction fragment length polymorphisms (Soller et al. 1976; Stuber et al. 1980; Tanksley et al. 1982). Applications of such inference techniques were marker-assisted backcross selection schemes (Hospital et al. 1992), which allowed faster and more precise introgression of major QTL for phenotypes like disease resistance and morphological traits (Breeding 3.1). QTL models, initially designed for analyzing few genetic loci, were then extended to genome-wide models incorporating information about many genetic markers across the genome, such as genomic prediction models (Meuwissen et al. 2001). In major crops like maize and rice, using genomic prediction approaches has become common practice to compare and select the best individuals for complex traits (Heslot et al. 2015; Bevan et al. 2017), while genome-wide association mapping has successfully identified QTL for relatively simple traits related to flowering and leaf architecture (Huang and Han 2014) (Breeding 3.2). As genotyping and phenotyping technologies become cheaper, breeders will likely expand these techniques to new crops and in smaller breeding programs. Even though Breeding 3 began nearly 30 years ago, the use of genomics in crop improvement is far from widespread across species and regions of the globe. Future plant breeding will expand Breeding 3 globally and will begin the transition to Breeding 4 (Fig. 1).

## Statistical limitations in plant breeding and the transition to Breeding 4

### The concept of causal loci and the curse of dimensionality

**Fig. 2:**
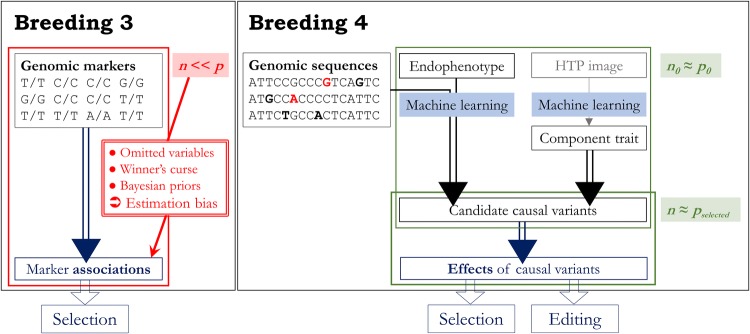
The shift in statistical framework between Breeding 3 and Breeding 4. Breeding 4 will aim to avoid the curse of dimensionality (*n* ≪ *p*) which precludes the inference of causal loci, especially for traits controlled by many causal loci. Machine learning techniques such as neural networks will be useful under this novel framework, to model effects of DNA polymorphisms on endophenotypes and predict component traits from high-throughput phenotyping (HTP) data

The concept of loci controlling agronomic traits has been at the core of breeders’ ability to describe and exploit genetic variability. As breeding science progressed, a better understanding of causal loci has increased breeders’ control over the genetics of complex traits. In Breeding 1, the basis for variability at agronomic traits was unknown, making mass selection the only viable breeding scheme. In Breeding 2, the concept of a causal locus (“Mendelian factor”) allowed breeders to exploit family relationships and more accurately estimate breeding values (Fisher 1919; Wright 1921). Nevertheless, this concept was still a theoretical construct and assumed an infinitesimal model for complex traits: it could not account for deviations from expected genetic relationships due to finite numbers of causal loci and differential effects across loci. Breeding 3 addressed these limitations through marker technology and linear regression models. In Breeding 3, genetic markers represent causal loci as QTL, allowing their contribution to phenotypic variability to be depicted explicitly. However, these innovations introduced both technological and statistical challenges. Due to ascertainment bias (sampling bias of markers during their development), genetic markers in Breeding 3 generally do not assay the whole pool of DNA polymorphisms in any given population (Heslot et al. 2013). Furthermore, utilizing marker information typically implies estimating effects on many more loci (*p* genotypic variables) than there are observations *at the genotype level* (*n* plant genotypes, i.e., instances with distinct genetics): *n* ≪ *p*. As a result, model parameters cannot be solved without simplifying assumptions. So, even though Breeding 3 models may accurately predict genetic merit, they should not be used to infer effects of many QTL. This curse of dimensionality is a critical issue in Breeding 3, and it severely hampers applications based on estimated QTL effects, especially for complex traits. In models based on few markers, omitted marker information results in models that fail to account for the confounding effects of loci. For example, apparent QTL effects may be caused by unobserved causal loci that are physically linked to genetic markers. In marker screens involving single-marker analyses or stepwise model selections (e.g., association mapping analyses), estimated effects may fluctuate randomly around their true values. These random fluctuations can reach such extremes that the most significant estimates might be highly overestimated in magnitude: this is the winner’s curse, also known as the Beavis effect in plant breeding (Beavis 1998; Xu 2003). Finally, when *n* ≪ *p*, models fitting all marker effects simultaneously must make assumptions about their distribution (typically, a normal distribution around zero) by means of regularization, such that their estimates are distorted (Gianola 2013). All three aforementioned cases illustrate the common issue of estimation bias regarding QTL effects in an *n* ≪ *p* context, and therefore stress the inadequacy of observed marker associations to make causal inferences about complex traits in Breeding 3 (Fig. 2).

The defining goal of Breeding 4 is to alleviate the issues of ascertainment bias and high dimensionality (*n* ≪ *p*) in order to make reliable inferences about numerous effects of loci. More exhaustive assays of genetic variability, using whole-genome or haplotype sequence data, will allow scientists to directly query the effects of observed loci, instead of estimating them indirectly through linked genetic markers. Furthermore, analyses involving higher *n* and/or lower *p* will permit more reliable inference of causal loci. This new framework will make use of sequence or image data and will therefore benefit from innovative machine learning approaches, different from the linear regression models typically used in Breeding 3 (e.g., see Ben-Hur et al. 2008 and Angermueller et al. 2016 for general introductions to such techniques in computational biology). Inference of causal loci will be key to define plausible plant ideotypes for guiding biological designs of improved varieties.

### The statistical and technological relevance of Breeding 4

While Breeding 3 used marker associations to make selections, Breeding 4 will efficiently integrate genotype and phenotype data to identify causal genetic features for breeders to make selections and perform biological interventions based on genetic transformation technologies (Wallace et al. 2018). Already such technologies have been used to transfer individual genes into plant varieties or to make precise edits at key genes controlling high-value traits (Breeding 4.1). For example, transgenic modification has been used to introduce insect resistance traits into maize cultivars (Koziel et al. 1993) or reduce browning in apple (Armstrong and Lane 2013), and gene editing by clustered regularly interspaced short palindromic repeats (CRISPR) has already produced useful characteristics by modifying single genes, like ARGOS8 for increased drought tolerance in maize (Shi et al. 2017). Continued research in gene editing is paving the way to parallel editing across the genome, as was demonstrated by methods for simultaneous gene editing of 16,006 sites in yeast samples (Sharon et al. 2018). Future iterations of the CRISPR technology will likely make it possible to modify complex traits in plants with thousands of directed edits (Breeding 4.2). Improvements in genetic transformation techniques will further facilitate gene transfer and gene editing, particularly in grass species (Lowe et al. 2016), making parallel gene editing all the more practical as a crop improvement tool. Compared to marker-assisted breeding, editing will create exciting new possibilities. It will accelerate the breeding cycle and reduce reliance on random recombination events for genetic improvement. It will also allow breeders to shorten the breeding process because, contrary to multi-parental programs in Breeding 3, combining the best polymorphisms from many plant genotypes will not require more crosses. Gene editing will allow the development of cultivars with fewer deleterious mutations, both in low-recombination regions where genetic burden has accumulated due to low crossover rate and genetic hitchhiking, and in high-recombination regions where they are difficult to purge via recombination due to their close proximity to beneficial alleles (Rodgers-Melnick et al. 2015; Ramu et al. 2017).

QTL models in Breeding 3 commonly rely on linear relationships between genetic markers and agronomic traits. Linear mixed models and related frameworks have been widely used to predict agronomic traits in genomic prediction (Meuwissen et al. 2001) and identify QTL in association mapping (Yu et al. 2006). These models have successfully guided breeding decisions; however, the next phase of plant breeding, Breeding 4, will aim to estimate locus effects accurately, not only to predict breeding values but also to infer causal loci (Fig. 2). Therefore, Breeding 4 will have to either reduce *p* or increase *n*. High-throughput phenotyping on simpler component traits (morphological and physiological traits) can be used to produce data on more genotypes, or endophenotypes (intermediate molecular traits such as gene expression levels or metabolic activity) can be used to provide many phenotypes per genotype (e.g., using expression data on every gene in a genotype, as in Kremling et al. 2018). Alternatively, the number of loci assayed can be decreased by focusing only on single genes or regions of the genome (e.g., focusing on polymorphisms in promoter regions and the UTR, as in Washburn et al. 2018). Relationships between polymorphisms and phenotypes can be depicted more accurately with genetically simpler traits than with the agronomic traits used in Breeding 3. In Breeding 4, using simpler endophenotypes or component traits as phenotypes should therefore reduce the required model complexity. Biologically meaningful models are critical, because Breeding 4 will require a significantly better understanding of the relationship between genotypes and phenotypes (the genotype–phenotype map) to define genetic ideotypes consisting of favorable alleles at causal loci for agronomic traits. This goal differs substantially from those in Breeding 3, where genetic data are used to mark loci rather than interrogate them directly, and will involve data types and modeling approaches that are complementary to those used in Breeding 3. Breeding 4 will use more functionally relevant features to infer causal loci (e.g., DNA sequence data rather than genetic markers) and will involve different models than simple linear regressions. Sophisticated pattern recognition algorithms will be critical in Breeding 4. Complex machine learning models will be needed to interrogate sequence data and capture nonlinear effects of polymorphisms on endophenotypes, or to estimate component traits based on high-throughput phenotyping image data (Fig. 2). These include nonlinear models such as neural networks, consisting of latent variables arranged in hidden layers (Bishop 1995). In Breeding 4, neural networks such as convolutional and recurrent neural networks (CNNs and RNNs, respectively) may be particularly appropriate, not only because they can capture nonlinear relationships to their output but also because they are designed to use sequence or image data as inputs. These models have been developed since the 1980s (LeCun et al. 1998; Deng et al. 2013), but their widespread applications to biological problems emerged much later, around 2014 (Angermueller et al. 2016), thanks to progress in computer hardware technology (LeCun et al. 2015) and critical improvements in optimization techniques for neural networks (e.g., Kingma and Ba 2014; Ioffe and Szegedy 2015).

## Using machine learning to prioritize causal loci in Breeding 4

### Detecting causal polymorphisms from sequence data

Starting from Sanger sequencing in the 1970s, sequencing technologies have progressed toward higher throughput and lower cost per nucleotide (Metzker 2005). The current state of sequencing technologies consists of short reads from next-generation sequencing, used together with less accurate but considerably longer reads from Pacific Biosciences or Oxford Nanopore Technologies sequencers (Goodwin et al. 2016). Improvements in sequencing technologies will make it easier to build and update reference genomes and assemblies, which will be useful for cataloging causal variants within their haplotype context in breeding populations. Haplotypes are small portions of chromosomes that are the basic units of heredity. They consist of DNA sequences which can be inferred based on marker data or low-depth, highly multiplexed sequencing data. Haplotype graphs are convenient computational frameworks to accurately infer haplotype sequences, as was made evident from studies in humans (Eggertsson et al. 2017). These graphs will provide a concise representation of genetic diversity from inexpensive genetic data, which should make Breeding 3 techniques (especially genomic prediction) accessible to breeders with low computer memory resources and limited genotyping budgets. In Breeding 4, haplotype graphs will provide fully contextualized DNA sequence information to genetic models. Such inputs should be more relevant depictions of the genetic bases of traits than genetic markers and should assay polymorphisms more thoroughly, hence remedying the issue of ascertainment bias. Furthermore, they will provide contextual information about possible effects of loci, e.g., a single nucleotide polymorphism (SNP) acting by disrupting a *cis*-regulatory motif. Finally, DNA sequences may be augmented with functional annotations at the level of single nucleotides such that input sequences include external information about their potential effects. Functional annotations may include characteristics about chromatin accessibility, which has been shown to account for a substantial amount of phenotypic variability in maize (Rodgers-Melnick et al. 2016), or information about selection pressure. For example, genomic evolutionary rate profiling (GERP) scores (Davydov et al. 2010) have been shown to correlate with SNP effects in maize (Yang et al. 2017). In general, haplotypes will contain detailed knowledge about nucleotides, genes and sub-chromosomal regions in a species, and should help extend genomic analyses to cross-species research, allowing breeders to use DNA information across species boundaries (Mace et al. 2013).

Breeding 1 and Breeding 2 relied on macroscopic traits to select the best individuals from a population. Breeding 3 has begun to use endophenotypes. Recent studies have combined transcriptome- and genome-wide association studies to increase power to detect causal genes (Kremling et al. 2018) and used metabolomic data to predict hybrid performance in maize (Schrag et al. 2018) and rice (Xu et al. 2016). Breeding 4 will extend the use of endophenotypes to precisely relate DNA information to endophenotypes and whole-plant performance. While suitable for analyzing additive effects of polymorphisms, typical Breeding 3 models are not equipped to reflect the effects of polymorphisms on endophenotypes in the context of particular DNA motifs. For example, they cannot accommodate differences in genomic position of functionally relevant patterns nor account for motifs occurring at multiple locations in a given genome. Moreover, these models cannot exploit biological replication within genotypes. While effects governing endophenotypes are likely similar, especially within a family of genes or regulatory regions, Breeding 3 models usually do not allow estimated effects to be shared across these similar contexts. Conversely, machine learning models have been designed to accommodate variability in occurrence of motifs and similarity in their effect across genomic regions. These include models based on counts or vector representations of *k*-mers in regulatory regions (Mejia-Guerra and Buckler 2017), as well as neural networks such as CNNs and RNNs which can recognize motifs occurring anywhere within sequences (by local scans in convolutions, or explicit dependencies in recurrences). Thus, machine learning can alleviate the limitations of Breeding 3 models and incorporate relevant information beyond genetic marker effects. Recent examples of machine learning applied to biological sequences include CNNs that use promoter sequences, coded as vectors of four binary variables (indicating each of the possible DNA nucleotides), to predict endophenotypes such as epigenetic marks (Angermueller et al. 2017) or transcription levels (Washburn et al. 2018; Zhou et al. 2018). Neural networks in these applications not only increased accuracy on prediction tasks involving DNA sequences, but also offered the possibility to prioritize variants based on their effects in the model, either by in silico mutagenesis (Zhou and Troyanskaya 2015) or by gradient computations (Washburn et al. 2018). Such prioritization based on endophenotypes holds great potential to enrich pools of polymorphisms for causal variants, as was recently suggested in humans (Zhou et al. 2018).

Another shortcoming of typical Breeding 3 models is their inability to easily accommodate equifinality issues: in QTL models, individual markers usually cannot represent the effect of multiple mutations causing the same phenotypes (e.g., different polymorphisms producing different STOP codons, all causing the coded protein to be non-functional). Differently, neural networks may capture biologically relevant effects of polymorphisms beyond additive effects and should be able to model effects of interactions between polymorphisms in local nonlinear functions (Poggio et al. 2017). In particular, locally connected neural networks (which include CNNs) may capture local epistatic effects within haplotypes, which are likely to be biologically meaningful (especially in genic regions) and inherited together (therefore contributing to additive genetic variance within a small breeding population). Therefore, machine learning analyses, complementary to Breeding 3, could increase power to detect causal variants of agronomic interest, because of important advantages: (1) they have the ability to use fully contextualized sequences as input data; (2) they can infer nonlinear relationships between polymorphisms and endophenotypes; and (3) they might be used in a *n* > *p* context, where inference about genetic effects might be possible (Fig. 2).

### Inferring morphological and physiological traits from image data

Most agronomic traits are controlled by complex physiological processes. Breaking complex traits into multiple phenotypic measurements may allow breeders to target different aspects of the trait, which is useful when breeding for consistency across environments (e.g., selecting for drought adaptation instead of directly for grain yield, which may depend on different adaptive traits depending on breeding environments; Cooper et al. 2009). Trait decomposition is all the more useful in marker-assisted breeding: splitting complex phenotypes into simpler component phenotypes also means splitting a highly quantitative trait into multiple traits with a simpler genetic basis (Hammer et al. 2005; Messina et al. 2011). However, direct assays of physiological traits (e.g., measurements of stomatal conductance or water-use efficiency) have critical disadvantages which preclude their utilization in breeding: (1) they are typically expensive and time-consuming; (2) they often must be performed in controlled conditions; and (3) they can be destructive. Such limitations can be overcome by imaging technologies which allow for high-throughput phenotyping by either rapid measurements of individual samples by field robots (Andrade-Sanchez et al. 2014), or single measurements of many samples at once by unmanned aerial vehicles (UAVs; Shi et al. 2016). Imaging technologies may capture radiation at different spectral ranges (visible range, near-infrared, far-infrared, etc.) with discrete or quasi-continuous wavelength resolutions, or other signals such as reflected laser pulses (LiDAR) which can be used to construct 3D images (Araus and Cairns 2014). Physiological and morphological traits can then be derived from images manually, e.g., normalized difference vegetation index (NDVI) calculated from reflectance at specific wavelengths (Sims and Gamon 2002), or photosynthetic rate calculated from chlorophyll fluorescence (Meyer and Genty 1998). Such derivation can also be automatic, however, rather than defined a priori and explicitly, and may thereby benefit from recent innovative machine learning approaches.

Traditionally, extracting trait information from image data on a single instance (e.g., an individual plant) has consisted of two steps: (1) segmentation, in which regions of interest for feature extraction are isolated (e.g., leaf area out of the background); and (2) analysis of structure, in which component traits are predicted from regions of interest (e.g., leaf counts from leaf area) (Spalding and Miller 2013). With the advent of neural networks, this framework may shift to a single processing step in which component traits are predicted directly from raw images, thereby moving away from trait-specific handcrafted predictors toward automatic abstract representations for predicting morphological and/or physiological traits (Singh et al. 2018). Examples of such approaches include CNN-like models for inferring leaf count from images of *Arabidopsis* plants (Giuffrida et al. 2018) or for classifying disease from leaf images in various plant species (Mohanty et al. 2016). Notably, DeChant et al. (2017) have shown the accuracy of CNNs for predicting northern leaf blight occurrence in maize from UAV images, demonstrating the applicability of neural networks for high-throughput phenotyping in the field.

While neural networks, especially CNNs, are promising for automatically predicting component traits from image data on a single instance, segmenting multiple instances from field images remains a challenge. As part of this effort, instance segmentation may be performed manually (Tsaftaris et al. 2016) or automatically, the latter being based on GPS coordinates (e.g., delineating plots in field images from UAVs; Shi et al. 2016) or pattern recognition. In particular, region-based CNNs have been used to simultaneously classify and isolate instances in global images (Girshick et al. 2014; He et al. 2017). One example comes from Jin et al. (2018) who used region-based CNNs to detect maize stems from field images.

Despite the advantages of automatic pattern recognition for image analysis, strategies based on neural networks for high-throughput phenotyping will face the challenge of interpretability inherent to the “black-box” nature of this type of models: predictions may depend on confounded factors rather than meaningful physiological or morphological characteristics. However, useful techniques exist to train models to be more robust to confounders, such as size or orientation of patterns in the image, e.g., data augmentation with shifted, re-scaled and/or rotated images (Bishop 2006). Hence, the combinations of machine learning models such as CNNs, and practices for robust training such as data augmentation should ensure reliable inference of component traits from high-throughput phenotyping images.

For each genotype, estimation of component traits would provide useful replication over time, as opposed to replication across plants. This type of replication should be useful to increase the prediction accuracy for component traits on each plant, making use of correlation of measurements over time to fit time-series functions to the data (e.g., logistic regression of plant height over time) for estimating component traits as parameters of such functions (e.g., a slope parameter reflecting growth rate) (van Eeuwijk et al. 2018). Nondestructive and accurate measurements of component traits by analysis of high-throughput phenotyping images, relying on useful machine learning approaches and possibly time-series analyses, should therefore allow breeders to test more plant genotypes in the field (higher *n*), with high enough accuracy for applications in Breeding 4.

With high enough *n*, quantitative genetics models may be used to predict component traits and detect genetic markers linked to their causal variants (Messina et al. 2011). In Breeding 3, component traits predicted from DNA information could be incorporated in genomic prediction models, thereby increasing their predictive ability for agronomic traits. Examples of this strategy are prediction analyses based on multivariate linear mixed models (Sun et al. 2017) or nonlinear crop growth models (Messina et al. 2018). In Breeding 4, markers or genomic regions (e.g., haplotypes) showing significant associations with component traits could be used to prioritize variants. In all likelihood, this strategy, similar to the preselection of variants based on endophenotypes, could effectively alleviate the *n* ≪ *p* issue for subsequent analyses on agronomic traits (Fig. 2).

### Moving from *n* ≪ *p* to *n* > *p* for testing variants and developing improved varieties

Ultimately, Breeding 4 will aim to detect causal variants as precisely as possible for subsequent breeding or editing, using approaches complementary to traditional QTL mapping techniques. This effort will rely on various types of data: DNA information (either in the form of genetic markers or DNA sequences), genomic annotation (on epigenetic status or evolutionary constraints) and phenotypic data, including both agronomic traits of interest and intermediate traits such as endophenotypes or component traits. Breeding 4 has become possible because of rapid progress in genotyping technologies (DNA sequencing, measurement of endophenotypes and inference of haplotypes) as well as phenotyping technologies (image acquisition and robotics). This phase will be characterized by a shift in focus from genetic marker data to well-annotated haplotype data. The shift from genetic markers to haplotypes will further reduce the cost of genomic prediction and help expand Breeding 3 to new crops and programs. In Breeding 4, it will allow contextualized sequence data to be used as inputs to models predicting endophenotypes. Fortuitously, Breeding 4 is concurrent with the development of machine learning methodologies, in particular neural networks such as CNNs or RNNs, which are appropriate for estimating the effects of polymorphisms on endophenotypes (from DNA sequences) or analyzing high-throughput phenotyping data for predicting component traits (from images). Realistic depiction of the genotype-phenotype map for these simpler traits will allow to estimate effects for prioritizing a handful of putative causal variants that are worth assessing in subsequent analyses on agronomic traits (Fig. 2). Two plant genotypes (e.g., inbred lines in maize) may differ from one another by tens of millions of genomic locations. This prioritization process will dramatically reduce the pool of variants to be tested, using either quantitative analyses (e.g., Breeding 3 methods) or transformation technologies (e.g., CRISPR gene editing). Following prioritization, a testing phase will define plant ideotypes according to which improved varieties will be developed, by selection or biological design. In maize, we envision the process of nominating thousands of edits, then editing nominated loci simultaneously in a homogeneous inbred background, to reduce the *n* ≪ *p* issue (with only thousands of loci segregating) and to eliminate confounding by physical linkage of loci. Then, with reasonable scale in field trials, effects of loci can be measured to define reliable plant ideotypes for developing improved varieties by genomic selection or genome editing.

#### Author contribution statement

GPR, SEJ and ESB all contributed to developing the article's outline, reviewing the literature and writing the manuscript.
